# Short peptide 803sp inhibits the inflammatory response induced by *Escherichia coli* accompanied by M2 polarization of macrophages

**DOI:** 10.3389/fcimb.2026.1876535

**Published:** 2026-08-20

**Authors:** Shuo Han, Junxi Jiang, Wei Duan, Lin Luo, Ning Du, Wanxiong Wu, Mingwei An, Gulaliya Ailibieke, Samah Attia Algharib, Junfeng Liu, Wanhe Luo

**Affiliations:** 1 Engineering Laboratory for Tarim Animal Diseases Diagnosis and Control, College of Animal Science and Technology, Tarim University, Alar, China; 2 College of Agriculture and Animal Husbandry Science and Technology, Tacheng Vocational And Technical College, Tacheng, China; 3 Instrumental Analysis Center, Tarim University, Alar, China; 4 Department of Clinical Pathology, Faculty of Veterinary Medicine, Benha University, Moshtohor, Toukh, Egypt

**Keywords:** 803sp, antibacterial peptides, *Escherichia coli*, inflammation, macrophage polarization, wound healing

## Abstract

**Introduction:**

Prolonged antibiotic use has led to increased bacterial resistance, making antibacterial peptides a promising alternative. We previously identified that the anti-apoptotic lncRNA-803 could encode short peptides, though their functions were unknown.

**Methods:**

In this study, we explored the novel peptide 803sp, derived from lncRNA-803, to understand its anti-inflammatory and bactericidal roles. We assessed its antibacterial properties, morphology, efficacy, and safety. Using an *Escherichia coli* (*E. coli*)-infected skin wound model, we evaluated histopathological changes, collagen deposition, and tissue remodeling, along with macrophage polarization markers and inflammatory cytokines.

**Results:**

Our results show that 803sp is highly effective against *E. coli*, is blood-compatible, and aids wound healing by reducing inflammation and hematoma, while promoting collagen and blood vessel formation. The application of 803sp is associated with the polarization of macrophages towards the M2 phenotype, characterized by increased CD206 expression and decreased CD80 expression. Consequently, a substantial population of M2 macrophages secretes elevated levels of the anti-inflammatory IL-10, whereas a smaller population of M1 macrophages secretes pro-inflammatory IL-6 and TNF-α.

**Discussion:**

Ultimately, 803sp enhances wound healing after *E. coli* infection through both bactericidal and anti-inflammatory pathways. The new antibacterial peptide 803sp effectively inhibits the growth of *E. coli*, making it a promising alternative to traditional antibiotics.

## 1 Introduction

Bacterial infections represent a significant threat to both human and animal health (Casanova and Abel, 2024). The issue of bacterial infections in chronic wounds is escalating in severity, underscoring the urgent need for innovative therapeutic strategies (Coluccio et al., 2024). Clinically, the infected area typically exhibits erythema, edema, increased temperature, pain, and functional impairment (Ferreira et al., 2025). Persistent infections impede wound healing, facilitating bacterial proliferation in the bloodstream and the subsequent release of toxins, which can trigger a systemic inflammatory response (Adib et al., 2022). Recurrent bacterial infections may result in tissue fibrosis and irreversible organ dysfunction. Currently, antibiotic therapy remains the primary treatment modality. However, the escalating problem of bacterial resistance is leading to increased treatment costs and rising mortality rates (Lyu et al., 2023; Li et al., 2024; Xu et al., 2025). Consequently, the development of novel antibacterial agents has become a critical area of research. In this context, antibacterial peptides (AMPs) have emerged as a promising alternative strategy, owing to their unique mechanism of membrane disruption, broad-spectrum antibacterial activity, and low propensity for inducing resistance (Girdhar et al., 2024).

AMPs constitute a class of host defense molecules that are evolutionarily conserved and have garnered significant attention due to their broad-spectrum activity and ability to circumvent traditional resistance mechanisms (Maron et al., 2022; Ji et al., 2024). While AMPs are typically transcribed from protein-coding genes, recent advancements in genomics have unveiled that long non-coding RNAs (lncRNAs) serve as a novel source of bioactive peptides (Yi et al., 2024). LncRNAs may harbor small open reading frames (sORFs) that encode functional peptides (Zhang, 2024). The peptides encoded by lncRNAs represent an emerging and underexplored therapeutic resource.

LncRNAs are RNA molecules exceeding 200 nucleotides in length, which were once considered non-functional transcriptional products (Núñez-Martínez and Recillas-Targa, 2022). However, they are now recognized as key regulators of various biological processes, including immune regulation and tissue repair. Notably, some lncRNAs contain sORFs capable of encoding functional peptides. For instance, it has been demonstrated that the peptide encoded by lncRNA HOXB-AS3 inhibits the growth of colon cancer (Huang et al., 2017). The short peptide encoded by lncRNA PINT87aa can inhibit the aging of hepatocellular carcinoma cells (Xiang et al., 2021). Despite these advancements, the functions of peptides encoded by lncRNAs remain insufficiently explored, particularly regarding their dual roles in directly eliminating microorganisms and facilitating wound healing. This indicates that the identification of endogenous peptides with antibacterial properties from lncRNAs may offer novel approaches for the treatment of infectious diseases.

In our prior research, we identified a lncRNA, designated as lncRNA-803, which demonstrated the capacity to inhibit cellular apoptosis (Han et al., 2024, Han et al., 2026). Computational predictions suggested that lncRNA-803 may encode multiple short peptides, several of which possess physicochemical properties akin to AMPs. However, the antimicrobial functionality of these short peptides remains unverified. Consequently, in this study, we synthesized a short peptide derived from lncRNA-803, referred to as lncRNA-803 encoded short peptide (803sp). The purity and molecular weight of 803sp were assessed. We then evaluated the antibacterial efficacy and safety profile of 803sp. Furthermore, we developed a murine skin wound model infected with *Escherichia coli* (*E. coli*) to examine the tissue-level wound healing effects of 803sp. At the molecular level, we quantified inflammatory cytokines and macrophage polarization markers to elucidate the mechanisms underlying the wound healing properties of 803sp. The findings will offer novel therapeutic strategies for the treatment of bacterial infections in skin wound healing.

## 2 Materials and methods

### 2.1 Institutional review board statement

The Institutional Animal Care and Use Committee at Tarim University approved the study’s animal approach and all experimental protocols involving the handling of mice, under the guidelines set forth by the Animal Science Academy of Tarim University’s Ethics Committee (Approval Code: PA20250326013, Approval Date: March 26, 2025).

### 2.2 Peptide, experimental animals and bacteria

The freeze-dried powder of 803sp (MCLGLFKRKKKSGK) was synthesized by PEPTIDESBANK Company (China). A total of 45 female Kunming mice, aged 6 weeks and weighing 18 to 20 grams, were provided by Tarim University Animal Experiment Station. The mice were randomly allocated into three distinct groups: a phosphate-buffered saline (PBS) control group, an *E. coli* infection group, and an *E. coli* + 803sp group. Throughout the experiment, they had unrestricted access to food and water. The *E. coli* isolates were provided by the Engineering Laboratory for Tarim Animal Diseases Diagnosis and Control (China).

### 2.3 Bioinformatics

The physicochemical properties of 803sp were predicted using the online software Expasy (https://web.expasy.org/protparam/). The potential of 803sp as an AMP was predicted using APD6 (https://aps.unmc.edu/AP/). The advanced structure of 803sp was predicted using the AlphaFold (https://alphafold.com/).

### 2.4 Transmission electron microscopy (TEM)

The structural analysis of 803sp in solution was conducted utilizing TEM (JEM-2100Plus, JEOL Ltd., Japan). A volume of 10 microliters of the 803sp solution was deposited onto a copper mesh and allowed to equilibrate for 5 minutes at ambient temperature. Excess solution was subsequently removed using filter paper. Thereafter, a 2% sodium phosphotungstate solution (G1870, Solarbio, China) was applied to the copper mesh and left for 2 minutes at room temperature. The sample was then dried using an incandescent lamp for a duration of 2 minutes before being transferred to the TEM for examination.

### 2.5 High performance liquid chromatography (HPLC)

The purity of 803sp powder was assessed via HPLC. The mobile phase A comprised 0.1% trifluoroacetic acid (T818781, Macklin, China) in water, while mobile phase B consisted of 0.1% trifluoroacetic acid (T818781, Macklin, China) in acetonitrile (A757670, Macklin, China). Chromatographic separation was achieved using a C18 column, with an injection volume of 15 μL, a column temperature maintained at 30 °C, and a flow rate of 1 mL/min. The total chromatographic run time was 20 minutes, with mobile phase B ranging from 18% to 38% and mobile phase A from 62% to 82%. The detection wavelength was set at 220 nm.

### 2.6 Mass spectrometry (MS)

To prepare a stock solution of 803sp powder at a concentration of 1.0 mg/mL, dissolve the powder in ultra-pure water. Subsequently, dilute this stock solution to a final concentration of 10 μg/mL using the initial mobile phase, which consists of 50% water and 50% acetonitrile with 0.1% formic acid (F809715, Macklin, China). Following filtration through a 0.22 μm nylon filter membrane, conduct analysis using the Waters ZQ2000 quadrupole mass spectrometer (Waters Corporation, Milford, MA, USA).

### 2.7 Absorbance of bacterial culture medium

The minimum inhibitory concentrations of 803sp against *E. coli* were determined employing the broth microdilution method. The wild-type strain of *E. coli* was standardized to a concentration of 5×10^5^ CFU/mL. A volume of 100 μL of the bacterial suspension was then dispensed into each well of a 96-well plate for further analysis. The 803sp powder was diluted in PBS to obtain concentrations ranging from 2 to 2048 μg/mL. Subsequently, 100 μL of the 803sp solution was added to each well of the 96-well plate, followed by incubation at 37 °C for 24 hours. The absorbance of each well was measured at 595 nm.

### 2.8 Inhibition zones

The inhibition zones of 803sp against *E. coli* was estimated. Briefly, 15 mL of agar medium was placed into an aseptic plate, and once the agar solidified, an additional 5 mL of agar medium was added, along with 0.1 mL of bacterial fluid that contained 1 × 10^6^ CFU/mL of *E. coli*. After it had been set, the holes were made in the firm agar using the proper straw. Next, 50 μL of 803sp solution were added, respectively. PBS was used as a control. The strain of *E. coli* were incubated for 24 h at 37 °C in an incubator with 5% CO_2_, after which the size of the inhibitory zones was measured and recorded.

### 2.9 Crystal violet staining

A total of 1 mL *E. coli* culture (1 × 10^6^ CFU/mL) was dispensed into each well of a 96-well plate. The samples were then treated with 803sp and incubated at 37 °C under static conditions. Following incubation, the wells were gently washed three times with PBS (G4202, Servicebio, China) to remove planktonic cells. Biofilms were stained using a 0.1% crystal violet solution (G1014, Servicebio, China) for a duration of 15 minutes. Subsequently, any residual crystal violet was eliminated through washing with PBS (G4202, Servicebio, China). To facilitate visualization of the biofilms, 1 milliliter of 95% ethanol (E801077, Macklin, China) was introduced into the wells. The optical density (OD) of the samples was then quantified using a multimode plate reader set to a wavelength of 595 nm.

### 2.10 Scanning electron microscopy (SEM)

The morphological alterations of *E. coli*, both treated with 803sp and untreated, were examined through SEM. The experimental protocol was as follows: an incubation of 256 μg/mL of 803sp with an *E. coli* suspension at a concentration of 1×10^6^ CFU/mL was conducted at 37 °C for 24 hours. Subsequently, the bacterial cells were fixed using 2.5% glutaraldehyde at 4 °C for 2 hours, followed by two washes, each lasting 15 minutes. Dehydration was then carried out using a series of ethanol solutions with increasing concentrations (30%, 50%, 70%, 90%, 95%, and 100%), each for 20 minutes. Following critical point drying and gold sputter coating, the samples were analyzed using SEM (APREO, Thermo Scientific, USA).

### 2.11 Hemolysis test

Fresh blood was collected from New Zealand white rabbits and transferred into a centrifuge tube containing sodium heparin (QX0012, Servicebio, China). The sample was centrifuged at 1500 rpm for 10 minutes, after which the resulting pellet was washed three times with PBS (G4202, Servicebio, China). The lower layer of red blood cells was then resuspended in PBS (G4202, Servicebio, China) to prepare a 2% red blood cell suspension. Subsequently, 200 μL of the 2% red blood cell suspension was transferred into a 1.5 mL centrifuge tube and treated with 803sp solutions at concentrations of 128, 256, 512, and 1024 μg/mL, respectively. A 2% red blood cell suspension treated with ultrapure water served as the positive control, while a suspension treated with PBS (G4202, Servicebio, China) acted as the negative control. The centrifuge tubes were incubated at 37 °C for 2 hours. Following incubation, the samples were centrifuged at 1500 rpm for 15 minutes, and hemolysis was assessed in the supernatant. A volume of 100 μL of the supernatant was extracted and transferred to a 96-well plate, with three duplicate wells for each treatment group. A multifunctional microplate reader was used to measure the absorbance at 540 nm. The degree of hemolysis is estimated using the following formula: Hemolysis percentage (%) = (Abs - Ab0)/(Abs100 - Abs0) × 100, where Abs, Ab0, and Abs100 are the absorbance of the test sample, the suspension treated with physiological saline, and ultrapure water, respectively.

### 2.12 Wound healing

Prior to the experimental procedures, the dorsal hair of each mouse was shaved. Following anesthesia, full-thickness excisional wounds were created on the dorsal surface of the mice. Subsequently, a suspension of the *E. coli* (10^8^ CFU/mL) was applied to the wound sites of mice in both the *E. coli* infection group and the *E. coli* + 803sp group, with a volume of 40 μL per wound. At 48 hours post-infection, the skin wounds of mice in the *E. coli* + 803sp group were topically administered with 62.5 µL of freshly prepared 803sp solution at a concentration of 4096 µg/mL (dissolved in sterile PBS), corresponding to a dose of 256 µg per wound. The treatment was performed once daily. Mice in the PBS group and *E. coli* infection group received an equal volume of sterile PBS following the same schedule. The skin wounds of mice in the PBS control group, the *E. coli* infection group and the *E. coli* + 803sp group were photographed and recorded. The trial completed when the wounds in the one group healed completely. The size of the wound area was examined using the ImageJ software (National Institutes of Health, USA). The percentage of the original wound (%) = (So-St/So) × 100% was used to determine the wound healing rate, where So denotes the size of the wound at the beginning and St denotes the size of the wound at the time of measurement.

### 2.13 Hematoxylin and eosin staining (HE), masson staining, and immunohistochemistry (IHC)

Hematoxylin and eosin staining was performed following established protocols. Subsequently, sterile scalpels were utilized to excise skin tissues with dimensions of 1 × 1 cm. The excised samples were rinsed three times with PBS and then immersed in 4% paraformaldehyde (G1101, Servicebio, China) for fixation. The fixed tissues underwent a series of processes including dehydration, clarification, and paraffin embedding. Tissue sections were stained with hematoxylin and eosin (G1076, Servicebio, China) to assess morphological changes associated with the inflammatory response. Additionally, Masson staining was employed to evaluate morphological alterations related to collagen. The tissue sections were further subjected to immunohistochemistry analysis using antibodies specific to mannose receptor C-type 1 (CD206) (GB113497, Servicebio, China), CD80 molecule (GB114055, Servicebio, China), tumor necrosis factor alpha (TNF-α) (GB115726, Servicebio, China), interleukin 6 (IL-6) (GB11117, Servicebio, China), and interleukin 10 (IL-10) (GB11108, Servicebio, China) to investigate alterations in macrophage polarization and the expression levels of proteins associated with inflammation. H&E-stained, Masson-stained and IHC-stained images were viewed and quantified using SlideViewer (3D HISTECH, Hungary) and ImageJ (National Institutes of Health, USA) softwares. All the slices were encoded with random numbers by personnel who were not involved in the scoring process. This ensured that the researchers conducting the quantitative analysis remained completely blinded to the allocation of the experimental group until the final statistical analysis was completed.

### 2.14 Western blot

Mouse skin tissues were subjected to protein extraction using RIPA lysis buffer (G2002, Servicebio, China), and protein concentrations were subsequently quantified employing the bicinchoninic acid (BCA) assay (G2026, Servicebio, China). Equivalent quantities of total protein (20 μg) were resolved via 10% sodium dodecyl sulfate–polyacrylamide gel electrophoresis (SDS-PAGE) and then transferred onto polyvinylidene fluoride (PVDF) membranes. The membranes were blocked with 5% skim milk at room temperature for a duration of 2 hours. This was followed by incubation with primary antibodies targeting GAPDH (Servicebio, GB15004), mannose receptor C-type 1 (CD206) (Servicebio, GB113497), CD80 molecule (CD80) (Servicebio, GB114055), tumor necrosis factor alpha (TNF-α) (Servicebio, GB115726), interleukin 6 (IL-6) (Servicebio, GB11117), and interleukin 10 (IL-10) (Servicebio, GB11108), in addition to horseradish peroxidase-conjugated goat anti-rabbit IgG (Servicebio, GB23303) at 4°C overnight. GAPDH levels were utilized as the loading control. Protein bands were visualized using an enhanced chemiluminescence detection system, and the optical density of each band was scanned and quantitatively analyzed using ImageJ software (National Institutes of Health, USA).

### 2.15 Statistical analysis

All experiments were conducted with three independent biological replicates (n=3), where each replicate represents a separate experimental run containing a cohort of 5 mice (total 15 mice per group). For histological assessment, 5 fields of view were randomly selected from each section as technical repetitions. The average value of each field was regarded as a biological data point for each subject to avoid pseudo-repetitions. For statistical comparisons between two distinct groups (PBS group vs. *E. coli* group; *E. coli* group and *E. coli* + 803sp group), after confirming that the data were normally distributed, an independent sample t-test were conducted in SPSS 19.0 (IBM, Armonk, NY, USA). For comparisons among multiple groups, one-way ANOVA analysis of variance was used, followed by Tukey *post hoc* test in SPSS 19.0 (IBM, Armonk, NY, USA). Data presented as mean ± standard deviation. Differences were considered statistically significant if *P* < 0.05.

## 3 Results

### 3.1 Physical and chemical properties of 803sp

**Figure 1:**
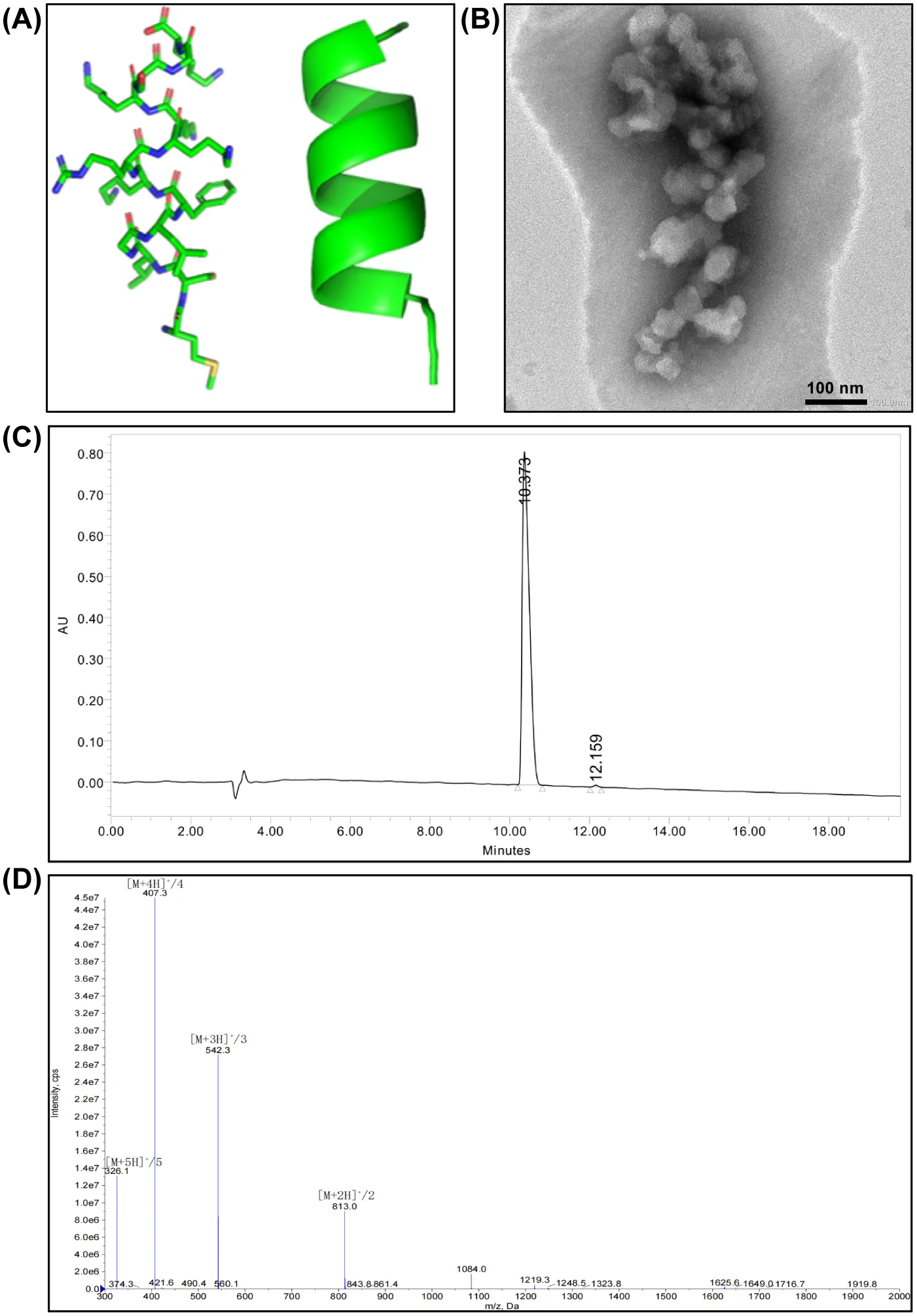
The physical and chemical properties of 803sp. **(A)** Predicted structure of 803sp based on bioinformatics. **(B)** Morphology of 803sp as observed via transmission electron microscopy. **(C)** Purity of 803sp analysed via HPLC. **(D)** Molecule weight of 803sp analysed via MS.

To gain a preliminary understanding of the characteristics and potential functions of 803sp, a comprehensive prediction analysis on its physicochemical properties were conducted. Our findings suggested that the molecular weight of 803sp was approximately 1.62 kDa. The compound was likely a cationic short peptide, potentially adopting an α-helical conformation, and might exhibit resistance to *E. coli* (**Figure 1A**). Subsequent microstructural analysis of 803sp was performed using TEM, which confirmed that 803sp might be a protein with an approximately α-helical structure (**Figure 1B**). To determine whether the purity of the synthesized 803sp met the standards, an HPLC test was conducted. HPLC analysis revealed a broad solvent peak at a low retention time (3 minutes), indicating a high degree of compatibility between the sample dissolution system and the mobile phase. The primary peak of 803sp, detected at an RT of 10.373 minutes, displayed a symmetrical monomodal profile, suggesting that the chromatographic separation conditions were well-optimized, with no loss of column efficiency or secondary interactions. The elevated absorbance unit (AU ≈ 0.8) of the principal peak, coupled with the lack of notable impurity peaks within the retention time (RT) interval of 4 to 9 minutes, corroborates the high purity and concentration of 803sp. Additionally, the considerable retention time disparity between the solvent peak and the principal peak (7.373 minutes) underscores the exceptional specificity of the method (**Figure 1C**). The molecular weight of 803sp was determined through an MS test. MS analysis revealed four distinct charge states for the compound 803sp: [M + 5H]^5^+, [M + 4H]^4^+, [M + 3H]³+, and [M + 2H]²+, corresponding to m/z values of 326.1, 407.3, 542.3, and 813.0, respectively. The molecular weight, derived from these charge states, was calculated to range from 1623.88 to 1625.46 Da, with a measurement error of ±0.8 Da, which is within the typical mass accuracy range of the instrument (<1 Da). The consistency observed across all charge states confirmed the molecular weight of 803sp to be approximately 1624.6 Da, which aligns closely with bioinformatics predictions (**Figure 1D**).

### 3.2 Antibacterial effect of 803sp on *E. coli*

**Figure 2:**
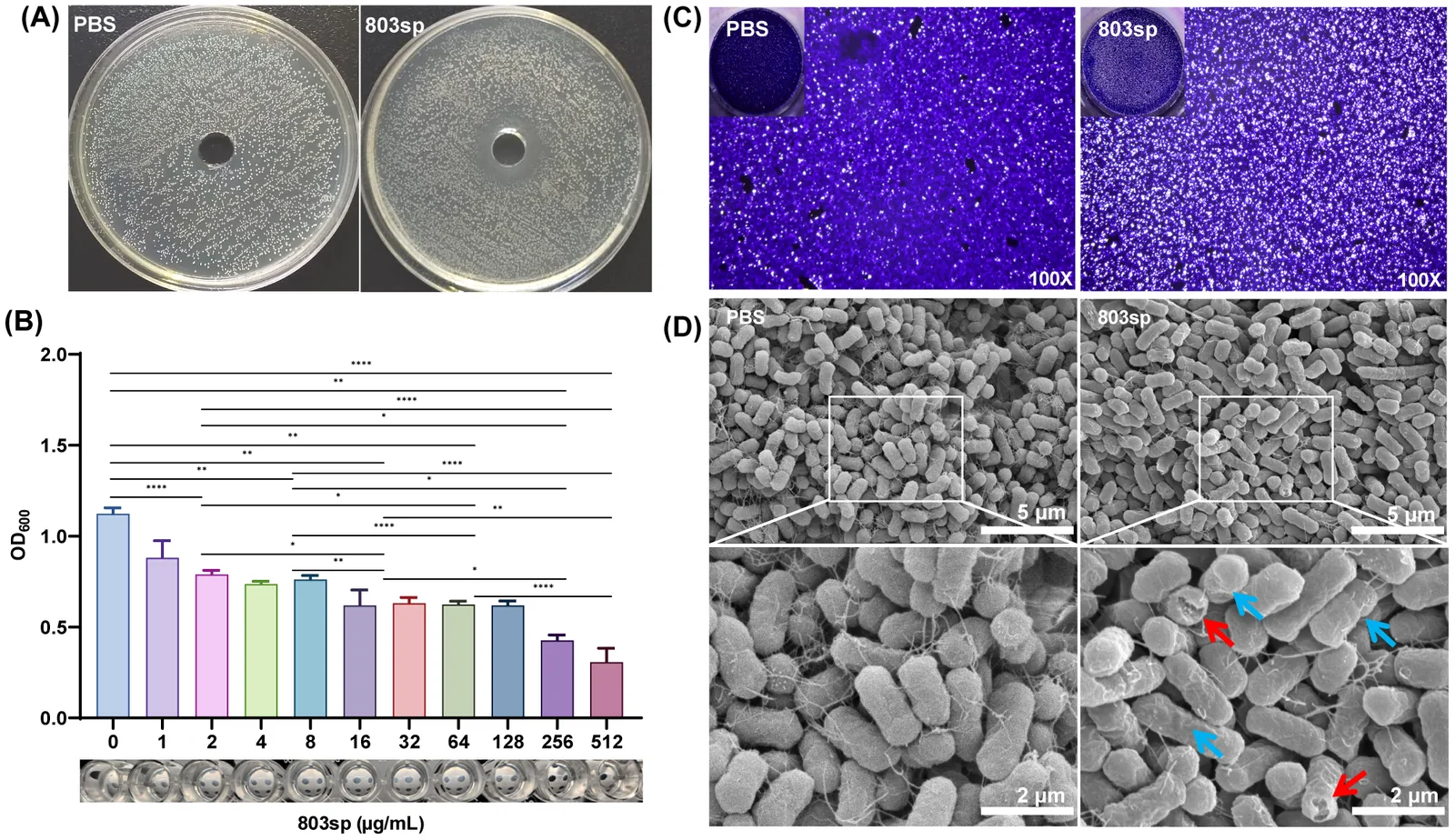
The antibacterial and anti-biofilm formation capabilities of 803sp. **(A)** The inhibition zone of 803sp against *Escherichia coli* (*E. coli*). **(B)** The inhibitory effect of 803sp on the growth of *E. coli*. The statistical analysis of various concentrations of 803sp treatment groups was performed using one-way ANOVA in SPSS 19.0 (IBM, Armonk, NY, USA). Data presented as mean ± standard deviation. Statistical significances between the various concentrations of 803sp treatment groups were indicated as follows: *P < 0.05, **P < 0.01, ***P < 0.001 and ****P < 0.0001. **(C)** The inhibitory effect of 803sp on the formation ability of biofilms in *E. coli*. **(D)** The morphologies of 803sp-treated and untreated *E. coli* observed by scanning electron microscopy. The red arrow indicates the damaged *E. coli*. The blue arrow indicates the wrinkled surface of the *E. coli*.

To evaluate the inhibitory efficacy of 803sp against *E. coli*, we measured the optical density values of the culture medium and assessed the inhibition zones. The findings demonstrated that 803sp significantly inhibited the growth of *E. coli* (**Figure 2A**). The minimal inhibit concentration of 803sp against *E. coli* is 256 μg/mL (**Figure 2B**). Additionally, the inhibitory impact of 803sp on biofilm formation was quantified using crystal violet staining. As depicted in **Figure 2C**, 803sp inhibits the initial stages of bacterial attachment and biofilm formation. Upon examination with scanning electron microscopy, *E. coli* treated with PBS demonstrated a characteristic rod-shaped morphology. Numerous slender and filamentous appendages were observed surrounding the bacterial cells, and the overall bacterial structure remained intact, exhibiting no apparent rupture or lysis. In contrast, following treatment with 803sp, the *E. coli* exhibited pronounced wrinkling, depressions, and deformation. Several bacterial cells displayed surface perforations and ruptures, and there was a marked reduction in the appendages surrounding the bacterial bodies (**Figure 2D**).

### 3.3 The *in vivo* safety of 803sp

**Figure 3:**
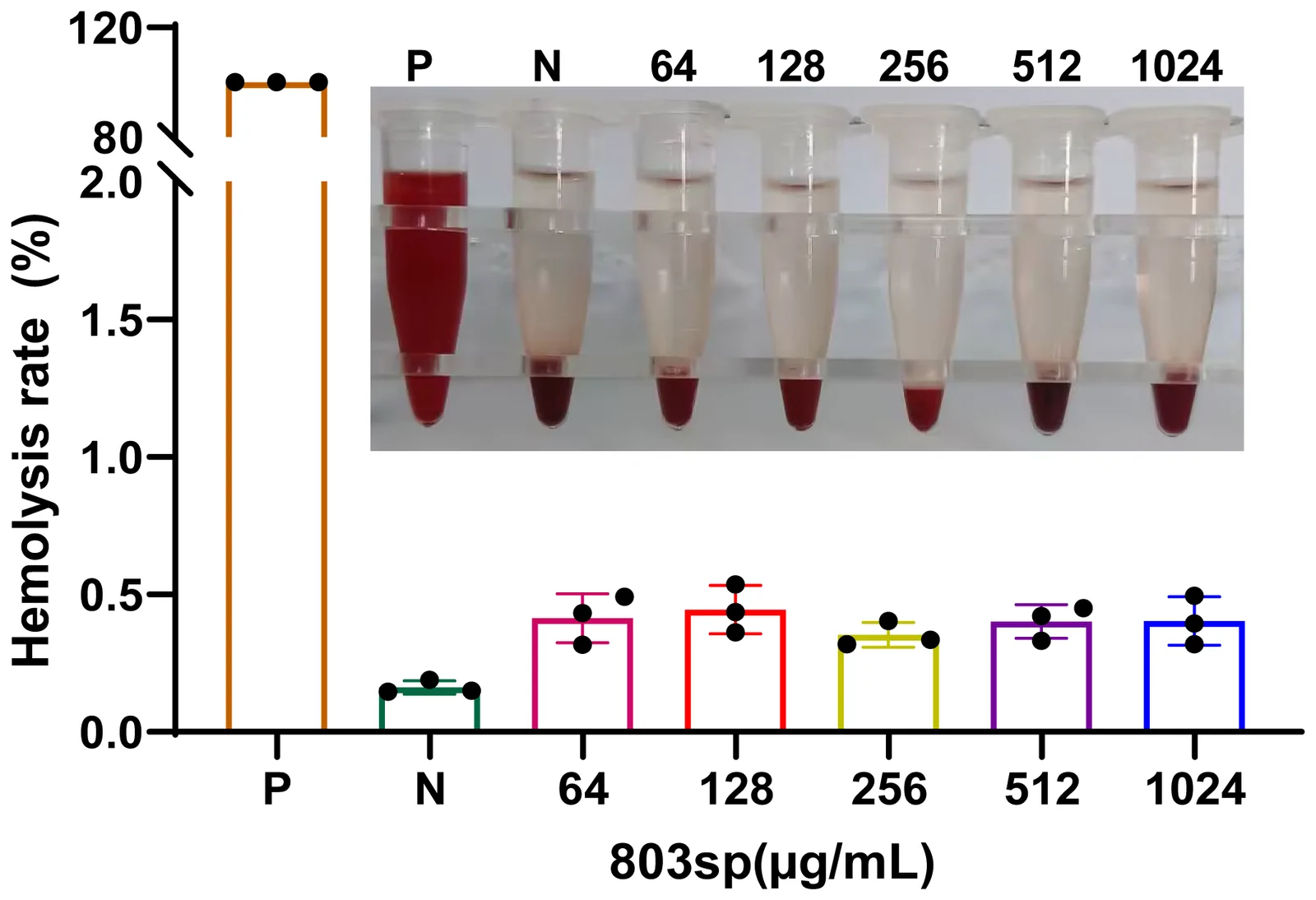
The *in vitro* biological safety of 803sp.

To further assess the safety profile of 803sp, rabbit blood was exposed to concentrations ranging from 64 to 1024 μg/mL of 803sp, with PBS serving as the negative control and water as the positive control. Hemolysis rates were subsequently measured. The findings indicated that the hemolysis rate for rabbit blood treated with 803sp at these concentrations remained below 1%, suggesting a favorable safety profile for 803sp (**Figure 3**).

### 3.4 The effect of 803sp on wound healing infected by *E. coli*

**Figure 4:**
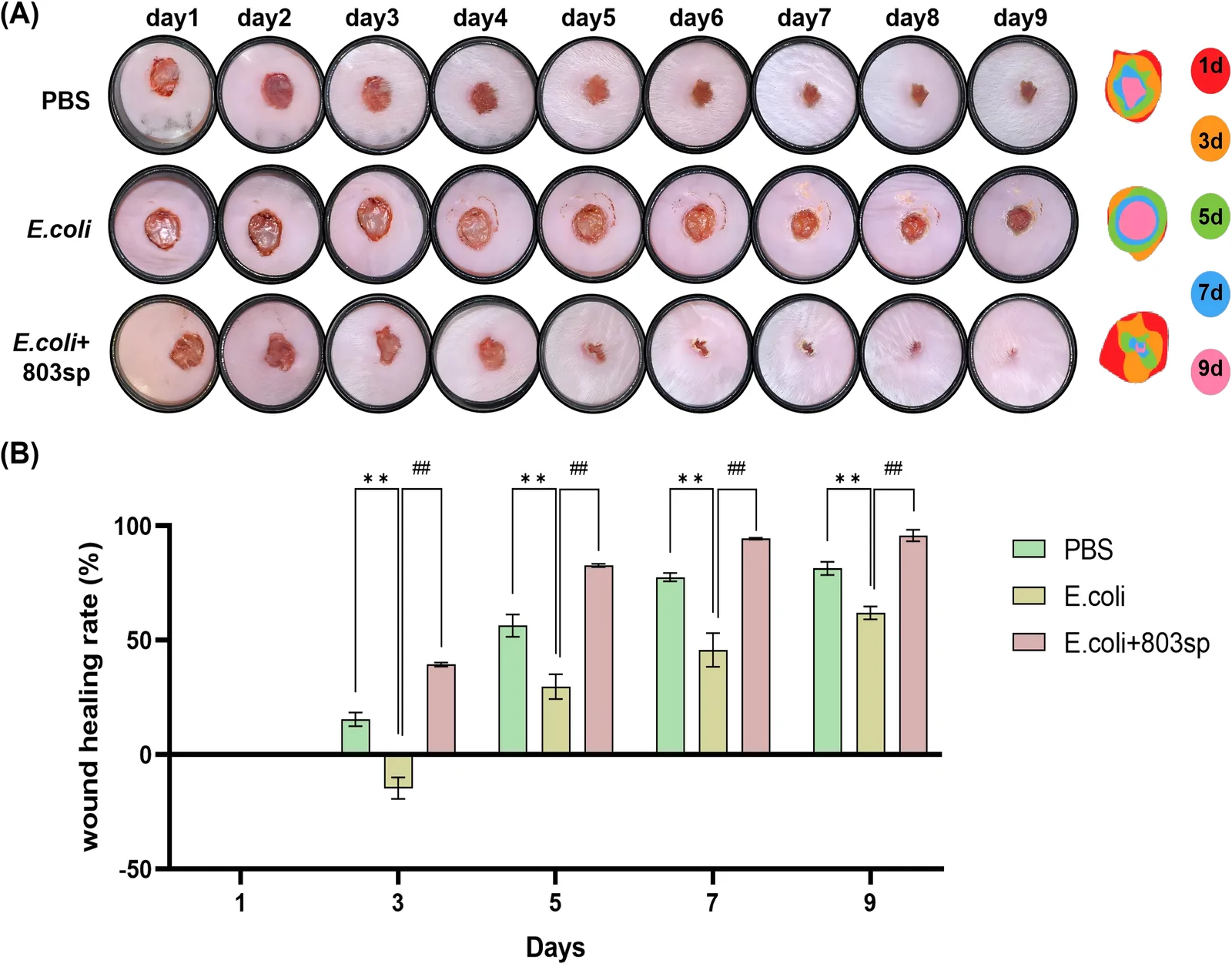
The effect of 803sp on wound healing after *E. coli* infection. **(A)** Representative images of *E. coli*-infected wounds in different treatment groups (PBS, *E. coli*, *E. coli* + 803sp) on day 1 to 9. **(B)** Corresponding wound healing rate recorded on days 1, 3, 5, 7, and 9. Statistical comparisons between two distinct groups (PBS group vs. *E. coli* group; *E. coli* group and *E. coli* + 803sp group) were conducted using the independent sample t-test in SPSS 19.0 (IBM, Armonk, NY, USA). Data presented as mean ± standard deviation. ** indicates *P* < 0.01, and there were an extreme significance between PBS group and *E. coli* group. ## indicates *P* < 0.01, and there were an extreme significance between the *E. coli* group and *E. coli* + 803sp group.

To evaluate the antibacterial and anti-inflammatory properties of 803sp *in vivo*, a murine skin wound model was established. The wounds were subjected to treatments with PBS, *E. coli*, and a combination of *E. coli* and 803sp. These treatments were categorized into control, *E. coli*, and 803sp groups. The healing progression of the wounds in each group was documented, healing trajectories were plotted, and wound healing rates were calculated. Representative images depicting the healing process of *E. coli*-infected wounds were also presented. The findings indicated that after nine days of treatment, wound healing was most delayed in the *E. coli* infection group, whereas the *E. coli* + 803sp group exhibited the most rapid healing. Notably, by the third day, *E. coli* infection resulted in a further enlargement of the wound area, suggesting that the infection induced skin inflammation and swelling. On days 3, 5, 7, and 9, the wound healing rate in the *E. coli* group was lower than that in the PBS group, whereas the *E. coli* + 803sp group demonstrated a significantly higher healing rate compared to the *E. coli* group (**Figure 4**). These results confirm that *E. coli* effectively infected the skin wounds in mice, causing swelling and extending the healing duration. Conversely, treatment with 803sp markedly accelerated the healing of skin wounds, indicating that 803sp possesses both antibacterial and anti-inflammatory properties.

### 3.5 Effect of 803sp on the histological changes of the skin tissues

**Figure 5:**
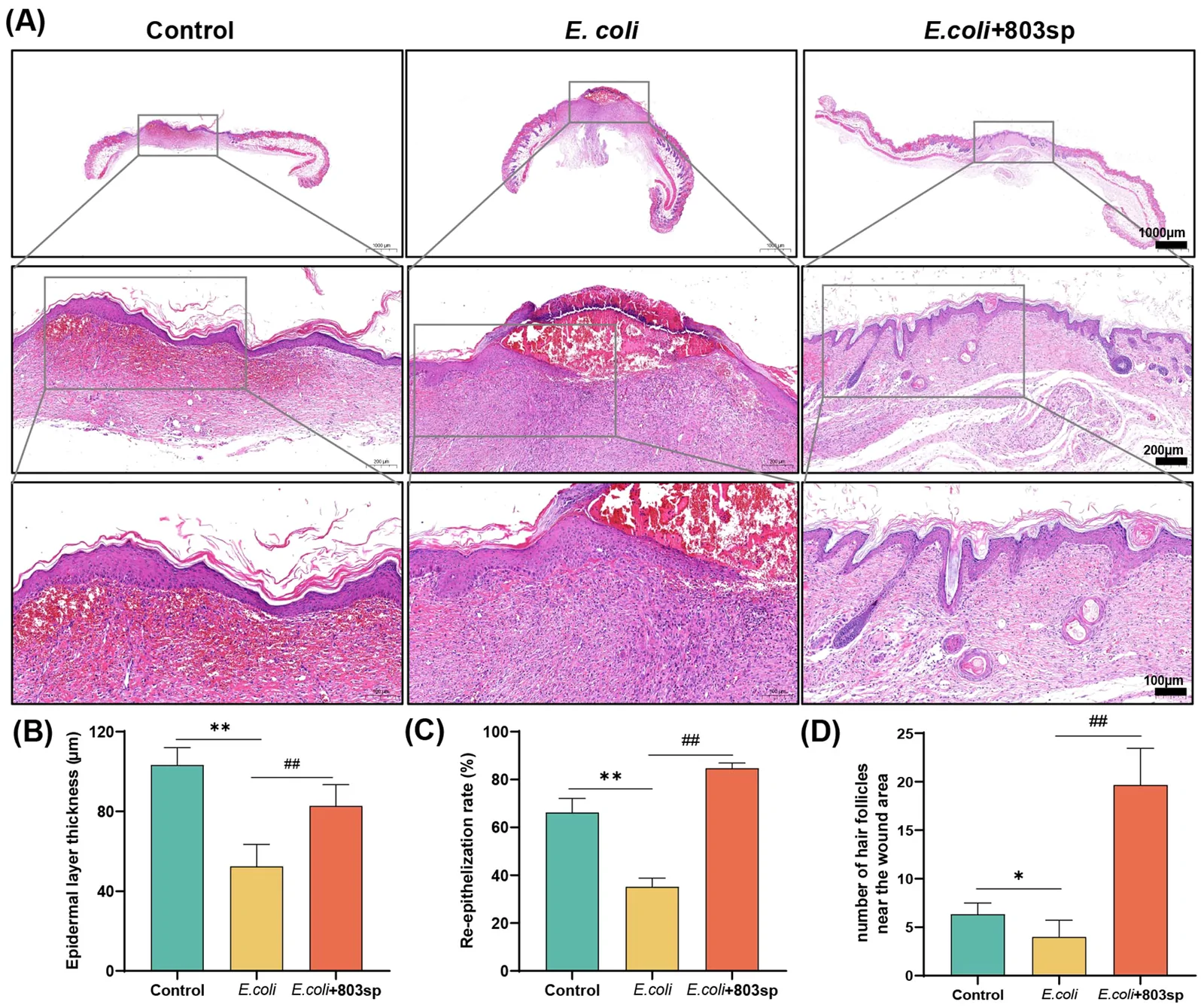
The repair effect of 803sp on skin wounds infected by *E. coli.* Note: **(A)** Representative images of pathological damage to skin wound tissue. Statistical results of epidermal layer thickness **(B)**, re-epithelialization rate **(C)**, number of hair follicles near the wound **(D)**. Statistical comparisons between two distinct groups (PBS group vs. *E. coli* group; *E. coli* group and *E. coli* + 803sp group) were conducted using the independent sample t-test in SPSS 19.0 (IBM, Armonk, NY, USA). Data presented as mean ± standard deviation. ** indicates *P* < 0.01, and there were an extreme significance between PBS group and *E. coli* group. ## indicates *P* < 0.01, and there were an extreme significance between the *E. coli* group and *E. coli* + 803sp group.

To further investigate the histological impact of 803sp on wound healing, we performed HE staining on skin wounds on the ninth day post-injury. In the PBS group, there was a notable infiltration of neutrophils and mast cells within the subcutaneous tissue, accompanied by a diffuse and uniform distribution of extravasated red blood cells. Despite this, a complete epidermal layer was observed, along with the presence of some hair follicles and sebaceous glands in proximity to the wound. In contrast, the *E. coli* infection group exhibited a significantly higher infiltration of inflammatory cells and red blood cells in the subcutaneous tissue compared to the PBS group. The wound surface was covered with scabs, and a hematoma was present between the scabs and the subcutaneous tissue. Only a limited number of hair follicles and sebaceous glands were observed near the wound in this group. In the *E. coli* + 803sp group, there was an absence of discernible infiltration by inflammatory cells or erythrocytes within the subcutaneous tissue, and a fully developed epidermal layer was observed. Numerous hair follicles and sebaceous glands were evident in proximity to the wound site. Statistical analyses revealed that the epidermal thickness, re-epithelialization rate, and hair follicle count in the *E. coli* infection cohort were significantly reduced compared to the PBS group. Conversely, these parameters were significantly elevated in the *E. coli* + 803sp group relative to the *E. coli* infection group (**Figure 5**). These findings suggest that 803sp effectively mitigates inflammatory cell and erythrocyte infiltration, facilitates the development of hair follicles and sebaceous glands, enhances epidermal layer formation, and consequently expedites the repair of cutaneous wounds.

### 3.6 Effect of 803sp on the deposition of collagen of the skin tissues

**Figure 6:**
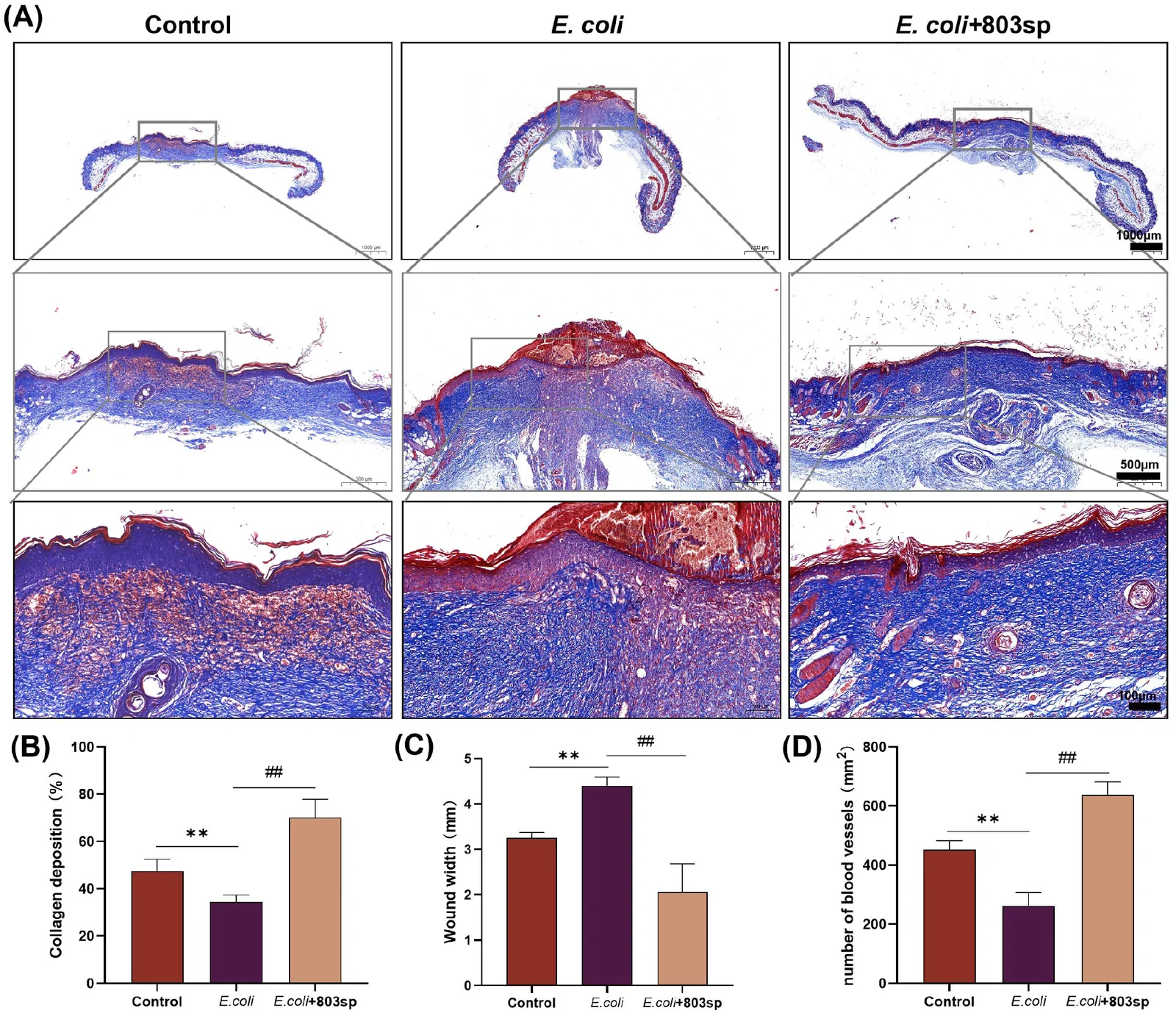
The effect of 803sp on collagen formation in skin wounds infected by *E. coli.* Note: **(A)** Representative images of collagen formation in skin wound tissue.Statistical results of collagen deposition **(B)**, wound width **(C)**, number of blood vessels **(D)**. Statistical comparisons between two distinct groups (PBS group vs. *E. coli* group; *E. coli* group and *E. coli* + 803sp group) were conducted using the independent sample t-test in SPSS 19.0 (IBM, Armonk, NY, USA). Data presented as mean ± standard deviation. ** indicates *P* < 0.01, and there were an extreme significance between PBS group and *E. coli* group. ## indicates *P* < 0.01, and there were an extreme significance between the *E. coli* group and *E. coli* + 803sp group.

The synthesis and deposition of collagen within granulation tissue are central to the process of wound healing. To assess collagen fiber characteristics and evaluate collagen deposition, maturation, and tissue remodeling during wound healing, Masson staining was performed on skin wounds on the ninth day post-injury. In the PBS group, collagen fibers were abundant and exhibited a progressively more organized arrangement, with a relatively large area of collagen deposition. Conversely, in the *E. coli* infection group, collagen deposition was inhibited, characterized by sparse, lightly colored collagen fibers that were predominantly disordered and filamentous in distribution. In the *E. coli* + 803sp group, collagen deposition was restored, with an increased area of deposition, thicker fibers, and a tendency towards regular arrangement. Statistical analysis revealed that the collagen deposition rate in the *E. coli* infection group was significantly lower than in the PBS group, whereas the rate in the *E. coli* + 803sp group was significantly higher than in the *E. coli* infection group. The change in wound width serves as an intuitive and macroscopic indicator for assessing wound healing. In comparison to the PBS group, the wound width in the *E. coli* infection group was markedly increased, suggesting that *E. coli* infection induced persistent inflammation, inadequate fibroblast activation, and inhibited collagen deposition, thereby impeding both tissue filling and contraction. Conversely, the wound width in the *E. coli* + 803sp group was significantly reduced compared to the *E. coli* infection group, indicating that 803sp effectively mitigates the infection and facilitates wound closure. Observations in the PBS group revealed uniform exudation of red blood cells, suggesting active angiogenesis in the subcutaneous tissue. In contrast, the *E. coli* infection group exhibited a significant reduction in the number of subcutaneous blood vessels when compared to the PBS group, with the presence of crusts and a substantial accumulation of red blood cells in the subcutaneous tissue, indicating that *E. coli* infection led to vessel rupture or severe leakage, culminating in hematoma formation. In comparison to the *E. coli* infection group, the *E. coli* + 803sp group exhibited a significant increase in the number of subcutaneous blood vessels, with no evident leakage of red blood cells in the subcutaneous region (**Figure 6**). These findings suggest that 803sp facilitates angiogenesis and the restoration of the capillary network. Consequently, the results indicate that 803sp may expedite wound closure by enhancing collagen deposition and promoting angiogenesis.

### 3.7 Effects of 803sp on macrophage polarization and inflammation

**Figure 7:**
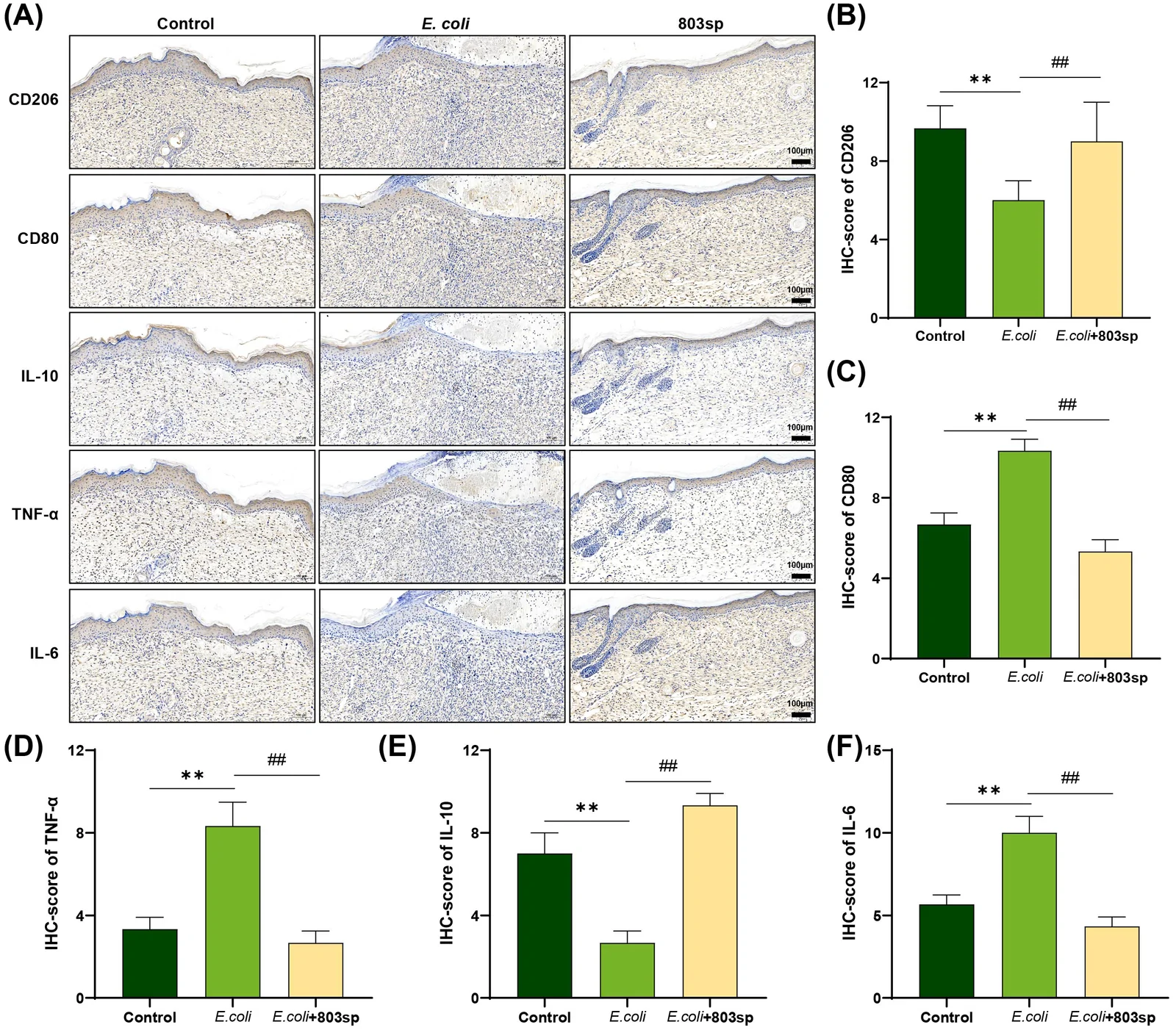
Observation results of the deposition of proteins related to macrophage polarization and inflammation in the skin wound tissue. **(A)** Representative image of immunohistochemical staining of proteins at the skin wound site. The statistical results of the IHC-score for CD206 **(B)**, CD80 **(C)**, TNF-α **(D)**, IL-10 **(E)**, and IL-6 **(F)**. Statistical comparisons between two distinct groups (PBS group vs. *E. coli* group; *E. coli* group and *E. coli* + 803sp group) were conducted using the independent sample t-test in SPSS 19.0 (IBM, Armonk, NY, USA). Data presented as mean ± standard deviation. **indicates *P* < 0.01, and there were an extreme significance between PBS group and *E. coli* group. ## indicates *P* < 0.01, and there were an extreme significance between the *E. coli* group and *E. coli* + 803sp group.

**Figure 8:**
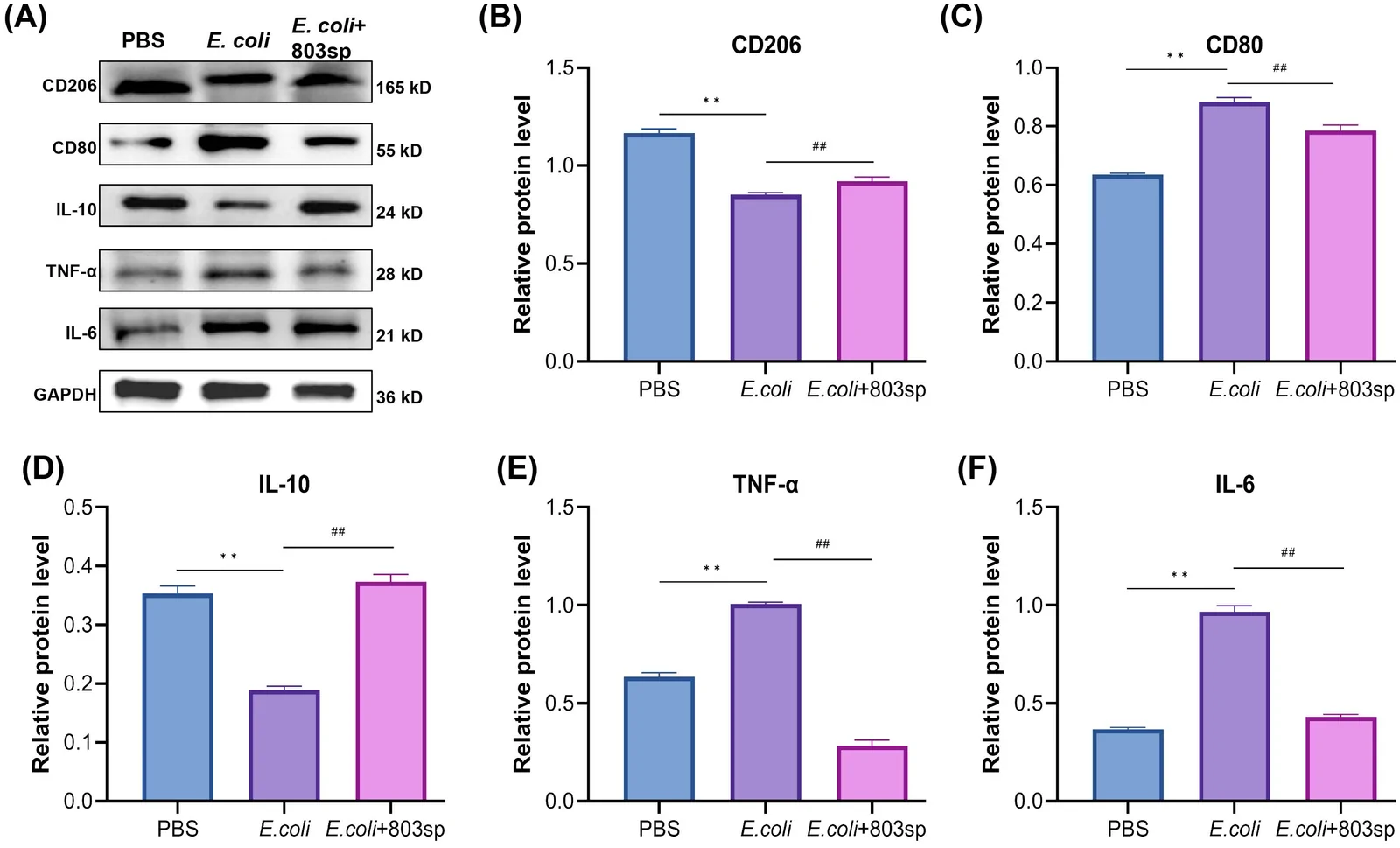
The Western Blot results of proteins related to macrophage polarization and inflammation in the skin wound tissue. **(A)** Representative image of the Western Blot for proteins at the skin wound site. Statistical results of relative protein expression levels for CD206 **(B)**, CD80 **(C)**, TNF-α **(D)**, IL-10 **(E)**, and IL-6 **(F)**. Statistical comparisons between two distinct groups (PBS group vs. *E. coli* group; *E. coli* group and *E. coli* + 803sp group) were conducted using the independent sample t-test in SPSS 19.0 (IBM, Armonk, NY, USA). Data presented as mean ± standard deviation. **indicates *P* < 0.01, and there were an extreme significance between PBS group and *E. coli* group. ## indicates *P* < 0.01, and there were an extreme significance between the *E. coli* group and *E. coli* + 803sp group.

Macrophages play a pivotal role as regulatory cells in the process of wound healing. The polarization states of macrophages, categorized as M1 or M2, are critical in determining whether skin healing progresses towards efficient repair or results in chronic inflammation. To assess whether the function of 803sp is related to macrophage polarization, we examined the expression levels of several key proteins in skin wounds. These proteins included CD206, a marker indicative of M2-type macrophages; CD80, a marker associated with M1-type macrophages; IL-10, an anti-inflammatory cytokine secreted by M2-type macrophages; and TNF-α and IL-6, pro-inflammatory cytokines secreted by M1-type macrophages. Immunohistochemical analysis (**Figure 7**) and Western Blot experiment (**Figure 8**) revealed that, in comparison to the PBS group, the *E. coli* infection group exhibited decreased levels of CD206 and IL-10, alongside increased levels of CD80, TNF-α, and IL-6. In contrast, after treated with 803sp, an increase in CD206 levels and a decrease in CD80 levels were observed, alongside elevated IL-10 and reduced TNF-α and IL-6 levels. Consequently, the immune microenvironment at the wound site is shifted from a pro-inflammatory state to one conducive to healing.

## 4 Discussion

Chronic wound infections, particularly those attributed to drug-resistant pathogens such as *E. coli*, pose a significant challenge in clinical settings (Mende et al., 2022; Zhou et al., 2025). The rise of multi-drug resistant bacteria necessitates the investigation of alternative therapeutic strategies, such as AMPs. AMPs are considered promising candidates due to their broad-spectrum antibacterial efficacy and minimal propensity to induce drug resistance (Bechinger and Gorr, 2017; Ajayakumar et al., 2022). Traditionally, AMPs are sourced from protein-coding genes (Song et al., 2023). Recent genomic advancements have identified that lncRNAs may contain sORFs encoding functional peptides (Li et al., 2025). These peptides encoded by lncRNAs represent a novel therapeutic resource yet to be fully developed. In light of the escalating issue of conventional antibiotic resistance, AMPs with novel mechanisms of action constitute a crucial area of research. This study has demonstrated that the peptide 803sp, derived from the anti-apoptotic lncRNA-803 (Han et al., 2024, Han et al., 2026), functions as an AMP with notable antibacterial and anti-inflammatory effects. It is worth considering that epitranscriptomic RNA modifications play a crucial role in the function of lncRNAs. If the sORF encoding 803sp in lncRNA-803 is modified or operates in a different environment, then the expression level and function of 803sp within the cell will be regulated (Eladl et al., 2026; Eladl and Estfanous, 2026). In contrast to conventional microbial sources or synthetically engineered AMPs, 803sp is an endogenous functional peptide encoded by a eukaryotic lncRNA, offering potential benefits such as being naturally derived and exhibiting low immunogenicity.

In this study, bioinformatic analyses predicted that 803sp is a cationic short peptide capable of forming an α-helix structure. This prediction was corroborated by direct observation of its approximate α-helix-like microstructure using electron microscopy. The α-helix is a characteristic functional structure of most natural AMPs, with the core active structures of known natural AMPs (Bao et al., 2024; Eladl, 2025a). This structural feature facilitates the electrostatic interaction between the positively charged amino acid residues of the short peptide and the negatively charged bacterial cell membrane. The insertion of the hydrophobic residues of AMP into the bacterial membrane can result in the leakage of bacterial contents, ultimately leading to bacterial cell death (Juretić and Simunić, 2019). The findings from bioinformatic predictions and electron microscopy observations collectively suggest the potential of 803sp to function as an AMP.

The HPLC analysis demonstrated that the synthesized 803sp molecules were uniform, undegraded, and free from aggregation. Additionally, the chromatographic conditions did not induce tailing or non-specific adsorption to the stationary phase. Subsequent MS analyses verified that the amino acid composition and sequence of 803sp precisely matched the designed target, with no deletions, insertions, or unexpected chemical modifications. This unequivocally confirmed the high purity of 803sp, ensuring that all subsequent observed biological effects could be attributed solely to 803sp.

*E. coli* is a common pathogen responsible for skin wound infections, urinary tract infections, and sepsis (Russell et al., 2023). Predictive analyses of the antimicrobial potential of 803sp suggest that *E. coli* is its target bacterium. Consequently, we systematically demonstrated that 803sp exhibits significant antibacterial activity against *E. coli*. Furthermore, we discovered that 803sp is capable of inhibiting the initial stages of biofilm formation of *E. coli*, which are a major factor contributing to bacterial resistance and chronic infections (Hurlow et al., 2025). Many conventional antibiotics exhibit limited efficacy against bacteria residing within biofilms, primarily due to the ability of biofilms to impede drug penetration and induce a dormant state in the bacteria (Ruhal and Kataria, 2021; Yao et al., 2022; Liu et al., 2024). At the same time, we acknowledge the necessity to investigate whether 803sp can actively disrupt the already formed biofilms within a mature biofilm model. Coupled with prior electron microscopy evidence confirming that 803sp possesses an α-helical structure, these antibacterial findings imply that 803sp may integrate into the bacterial cell membrane via its helical configuration, resulting in the leakage of cellular contents. While our current study primarily focused on the functional characterization of 803sp, future investigations employing advanced structural biology techniques—particularly in-cell nuclear magnetic resonance and cryo-electron microscopy—will be essential to elucidate the detailed conformational dynamics of both the parent lncRNA-803 and its derived peptide within the intracellular environment (Eladl et al, 2025; Eladl, 2025b).

In this study, across a broad concentration range of 64-1024 μg/mL, the hemolysis rate of rabbit erythrocytes exposed to 803sp remained below 1%. It suggest that 803sp does not compromise erythrocyte membrane integrity, even at elevated concentrations. The superior hemocompatibility of 803sp suggests its potential for inclusion in formulations such as wound dressings, hydrogels, and sprays, with minimal risk of harm to newly forming blood vessels or erythrocytes within the wound environment.

Some molecules exhibiting potent antibacterial activity *in vitro* might often experience diminished efficacy upon entering the body due to factors such as serum protein binding, protease degradation, tissue barriers, and interactions with the immune system. *E. coli* is a prevalent pathogen responsible for skin wound infections, particularly in chronic wounds and post-operative scenarios (Zhou et al., 2025; Hashem et al., 2023). In our study, utilizing a murine wound model, we successfully demonstrated that 803sp retains its efficacy in a realistic infection environment. Three days post-infection with *E. coli*, the skin wounds in the PBS group exhibited expansion, characterized by inflammation and swelling, whereas the E. coli+803sp group did not exhibit such deterioration. The observed reduction in wound swelling serves as a tangible indicator of inflammation mitigation (Zhao et al., 2016). This finding suggests that 803sp effectively eradicated the bacteria within the wound and prevented further exacerbation of the infection. Moreover, the wound healing rate in the *E. coli* + 803sp group was significantly accelerated compared to the *E. coli* infection group, achieving the fastest healing by the ninth day. Although 803sp did not completely eradicate all bacterial presence, it effectively mitigated the excessive inflammatory response induced by the infection.

Histologically, 803sp significantly reduced inflammatory cell infiltration and erythrocyte exudation associated with *E. coli* infection, thereby alleviating local hematoma and tissue damage. In mouse treated with 803sp, the wounds exhibited fully formed epidermis, in contrast to the *E. coli* infection group, which displayed only scabs and hematoma. Compared to the *E. coli* infection group, the epidermal layer in the *E. coli* + 803sp group was more intact, with a notable increase in epidermal thickness and re-epithelialization rate. These findings suggest that 803sp effectively accelerates epithelial reconstruction on the wound surface, which is crucial for wound closure, prevention of secondary infection, and fluid loss (Xue et al., 2022; Yin et al., 2022). This indicates that 803sp facilitates re-epithelialization rather than merely promoting wound contraction. In typical healing processes, fibrous scars are often formed, lacking the regeneration of skin appendages (Sorg and Sorg, 2023). The skin of mice treated with 803sp exhibited a significant presence of hair follicles and sebaceous glands, suggesting that the healing process was more akin to regeneration than mere repair, resulting in more comprehensive functional recovery. For patients suffering from burns, deep trauma, or chronic ulcers, mere wound closure is insufficient, as scars devoid of sweat glands or hair are susceptible to re-fracture (Dai et al., 2020). Traditional antibiotics primarily function to eradicate bacteria and, while they may indirectly reduce inflammation (Xiao et al., 2023). Traditional antibiotics generally do not actively facilitate the regeneration of hair follicles or sebaceous glands. In contrast, skin tissue treated with 803sp demonstrated an absence of significant inflammatory cell infiltration or red blood cell infiltration, indicating that 803sp effectively managed infection and excessive inflammation. Moreover, it preserved vascular integrity, thereby creating an optimal tissue microenvironment conducive to healing.

In clinical practice, bacterial infections frequently result in reduced collagen synthesis, compromised blood supply, and disrupted tissue remodeling (Rahim et al., 2017). The absence of adequate and well-organized collagen fibers hinders wounds from attaining tensile strength, making them susceptible to reopening (Zhu et al., 2021; Donovan et al., 2023). In the *E. coli* infection group, collagen deposition is sparse and exhibits a disordered filamentous configuration, suggesting that infection not only suppresses collagen synthesis but also disrupts normal tissue remodeling processes (Gardeazabal and Izeta, 2024). Conversely, in the *E. coli* + 803sp group, there is an increase in collagen deposition area, with fibers becoming thicker and more regularly arranged. This indicates that 803sp not only restores collagen quantity but also enhances collagen quality. The orderly arrangement of collagen is fundamental for the skin to regain tensile strength and flexibility (Benito-Martínez et al., 2025). Histological evidence directly demonstrates that 803sp can mitigate the collagen metabolic disorder induced by *E. coli* infection. During the wound healing process, fibroblasts differentiate into myofibroblasts, which facilitate wound contraction by drawing the wound edges closer together (Schuster et al., 2023; Younesi et al., 2024). Simultaneously, newly synthesized collagen and granulation tissue occupy the defect site. The 803sp enhances collagen deposition, facilitates tissue filling, reduces wound width, and accelerates the healing process. The formation of new blood vessels ensures the delivery of oxygen and nutrients to the healing tissue while also removing metabolic waste and pathogens (Mani et al., 2023). An adequate vascular network is fundamental for nutrient delivery, inflammation resolution, and tissue regeneration. In the absence of sufficient blood vessels, processes such as collagen synthesis, re-epithelialization, and accessory tissue regeneration are impeded. In the *E. coli* infection group, there was a significant reduction in the number of subcutaneous blood vessels, accompanied by substantial red blood cell leakage and hematoma formation, suggesting that the infection compromised the vascular structure, leading to local ischemia and microcirculatory dysfunction. Conversely, the *E. coli* + 803sp group exhibited a significant increase in subcutaneous blood vessels, with no notable red blood cell leakage, indicating that 803sp not only protected the vasculature from infection-induced damage but also actively promoted angiogenesis and the restoration of a functional capillary network. The 803sp not only exhibits antibacterial properties but also concurrently inhibits excessive inflammation, promotes collagen deposition and remodeling, enhances angiogenesis, and accelerates wound contraction, thereby synergistically facilitating wound healing through multiple pathways.

Macrophages play a pivotal role as the primary regulators of wound healing (Hassanshahi et al., 2022; Raja et al., 2025). M1 macrophages are crucial for pathogen elimination during the initial stages of infection (Kovaleva et al., 2025). However, their prolonged presence can result in chronic inflammation and tissue damage (Luo et al., 2024). Conversely, M2 macrophages are instrumental in clearing apoptotic cells, promoting angiogenesis, activating fibroblasts, and facilitating collagen deposition, thus driving the healing process (Louiselle et al., 2021). In skin infected with *E. coli*, there is an upregulation of CD80 expression, along with increased levels of TNF-α and IL-6, while CD206 expression and IL-10 levels are downregulated. The findings suggest that the infection commandeers the immune system, prompting macrophages to transition to the M1 state (Aitcheson et al., 2021). This evidence corroborates that *E. coli* infection sustains the wound in an M1 pro-inflammatory microenvironment over an extended period, thereby inducing persistent inflammation and causing damage to blood vessels and collagen deposition (Liu et al., 2020). Following the administration of 803sp, there is a noted decrease in the expression levels of CD80, TNF-α, and IL-6, alongside an increase in the expression levels of CD206 and IL-10. This suggests that 803sp is associated with the transition of macrophages from the M1 to the M2 phenotype. However, the causal relationship between these events remains to be established. Further mechanistic studies, including macrophage depletion and M2 polarization inhibition, are warranted to test whether M2 polarization is functionally required for the anti-inflammatory effect. M2 macrophages are pivotal in driving regenerative healing (Meng et al., 2025). Corroborating histological observations, an increase in M2 macrophage numbers may stimulate fibroblasts through the secretion of growth factors, leading to the synthesis of regularly aligned collagen, as evidenced by Masson staining. This process facilitates the proliferation of vascular endothelial cells, contributing to neovascularization, as indicated by an increased vascular count. Furthermore, it supports epithelial and hair follicle stem cells, thereby promoting re-epithelialization and appendage regeneration, as demonstrated by HE staining.

**Figure 9:**
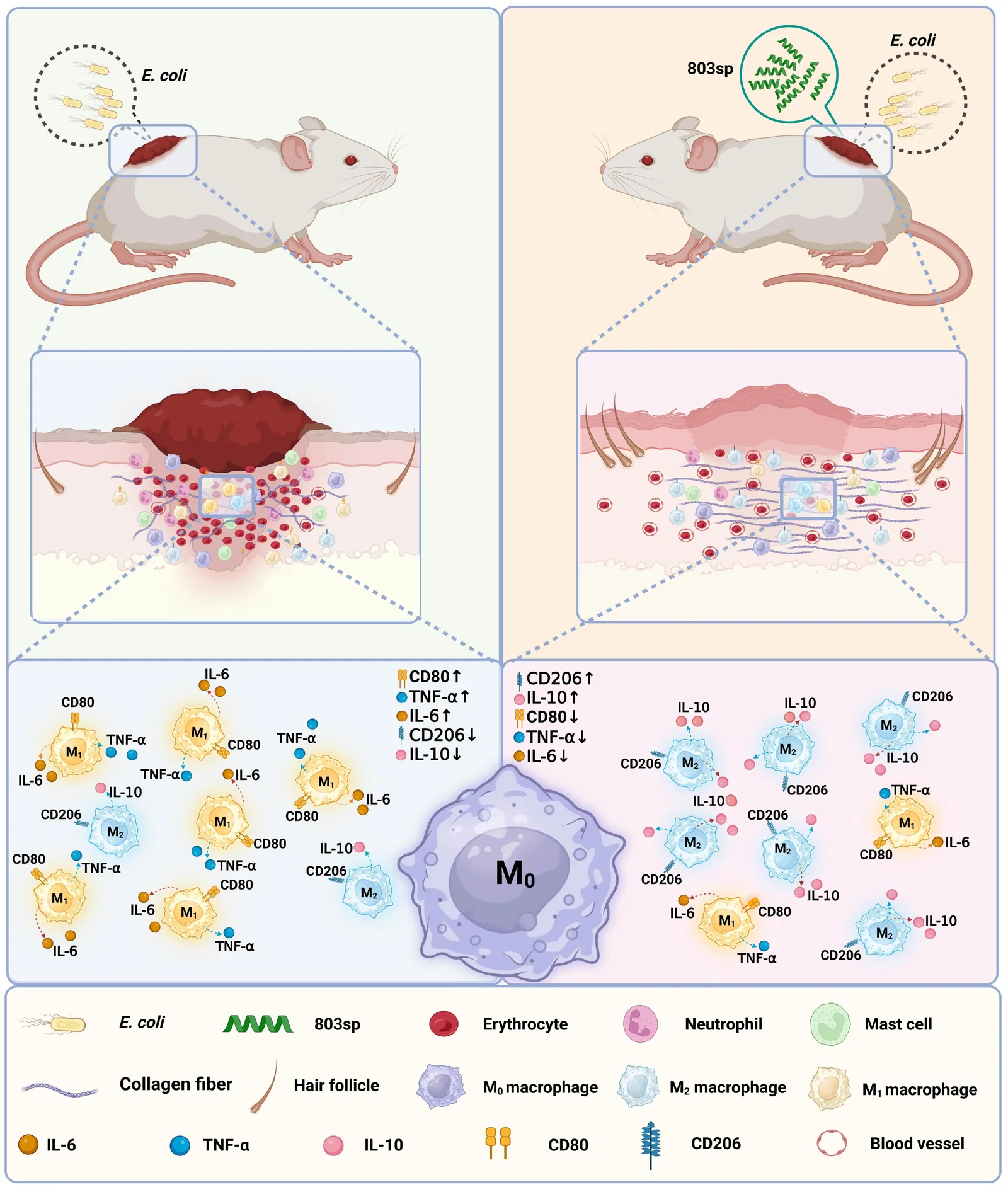
Schematic diagram illustrating the mechanism by which 803sp promotes wound healing in the context of *E. coli* infection.

This study identifies a peptide, 803sp, with strong antibacterial and immunomodulatory effects. It inhibits *E. coli* growth and biofilm formation, aiding infected skin wound healing. Topical 803sp accelerates wound closure, enhances healing, reduces wound width, decreases inflammatory and red blood cell infiltration, supports epidermis formation, and promotes hair follicle and sebaceous gland regeneration. It also boosts collagen deposition and arrangement. At the molecular level, these phenomena are accompanied by low expression of M1 polarization-related proteins, which reduces pro-inflammatory levels of TNF-α and IL-6, and high expression of M2 polarization-related proteins, which increases anti-inflammatory levels of IL-10 (**Figure 9**).

## 5 Conclusion

This study elucidates the pathways through which 803sp reduces inflammation and aids repair by balancing M1/M2 macrophages. The compound 803sp exhibits a range of functions, such as direct antibacterial activity, anti-biofilm properties, low hemolytic toxicity, and the ability to modulate macrophage polarization. These attributes enable it to effectively counteract immune imbalances and tissue repair disorders induced by *E. coli* infection. This research provides a robust theoretical framework and experimental foundation for the development of immune-regulatory antibacterial candidate drugs aimed at treating infected skin wounds.

## Data Availability

The original contributions presented in the study are included in the article/supplementary material. Further inquiries can be directed to the corresponding authors.

## References

[B1] Adib Y. Bensussan A. Michel L. (2022). Cutaneous wound healing: A review about innate immune response and current therapeutic applications. Mediators Inflamm. 2022, 5344085. doi: 10.1155/2022/5344085 35509434 PMC9061066

[B2] Aitcheson S. M. Frentiu F. D. Hurn S. E. Edwards K. Murray R. Z. (2021). Skin wound healing: Normal macrophage function and macrophage dysfunction in diabetic wounds. Molecules 26, 4917. doi: 10.3390/molecules26164917 34443506 PMC8398285

[B3] Ajayakumar N. Narayanan P. Anitha A. K. Mahendran K. R. Kumar K. S. (2022). Membrane disruptive action of cationic antibacterial peptide B1CTcu3. ChemBioChem 23, e202200239. doi: 10.1002/cbic.202200239 35713298

[B4] Bao R. Ma Z. Stanford K. McAllister T. A. Niu Y. D. (2024). Antimicrobial activities of α-helix and β-sheet peptides against the major bovine respiratory disease agent, Mannheimia haemolytica. Int. J. Mol. Sci. 25, 4164. doi: 10.3390/ijms25084164 38673750 PMC11050306

[B5] Bechinger B. Gorr S. U. (2017). Antimicrobial peptides: Mechanisms of action and resistance. J. Dent. Res. 96, 254–260. doi: 10.1177/0022034516679973 27872334 PMC5298395

[B6] Benito-Martínez S. Pérez-Köhler B. Rodríguez M. Rivas-Santos C. María Izco J. Recalde J. I. . (2025). Assessing new collagen therapies for wound healing: A murine model approach. Int. Wound J. 22, e70589. doi: 10.1111/iwj.70589 40258681 PMC12011449

[B7] Casanova J. L. Abel L. (2024). The microbe, the infection enigma, and the host. Annu. Rev. Microbiol. 78, 103–124. doi: 10.1146/annurev-micro-092123-022855 38986133 PMC11956784

[B8] Coluccio A. Lopez Palomera F. Spero M. A. (2024). Anaerobic bacteria in chronic wounds: Roles in disease, infection and treatment failure. Wound Repair Regen. 32, 840–857. doi: 10.1111/wrr.13208 39129662

[B9] Dai C. Shih S. Khachemoune A. (2020). Skin substitutes for acute and chronic wound healing: An updated review. J. Dermatolog Treat. 31, 639–648. doi: 10.1080/09546634.2018.1530443 30265595

[B10] Donovan C. Cogswell D. Sun M. Adams S. Avila M. Y. Margo C. E. . (2023). Collagen XII regulates stromal wound closure. Exp. Eye Res. 230, 109456. doi: 10.1016/j.exer.2023.109456 36967080 PMC10133200

[B11] Eladl O. (2025a). Biophysical and transcriptomic characterization of LL-37-derived antimicrobial peptide targeting multidrug-resistant Escherichia coli and ESKAPE pathogens. Sci. Rep. 15, 36126. doi: 10.1038/s41598-025-22890-7 41102328 PMC12533254

[B12] Eladl O. (2025b). In-cell NMR spectroscopy: Advancements, applications, challenges, and future directions in structural biology. Magn. Reson. Mater. Phys. Biol. Med. 39, 545–558. doi: 10.1007/s10334-025-01313-8 41428274

[B13] Eladl O. Abdelhady A. Tageldein M. A. (2026). Epitranscriptomic RNA modifications in human disease: Cooperative networks, molecular mechanisms, and therapeutic opportunities. Biochem. Biophys. Res. Commun. 815, 153673. doi: 10.1016/j.bbrc.2026.153673 41904917

[B14] Eladl O. Estfanous S. (2026). Beyond the obvious: Exploring the underappreciated dynamics and context-specific functions of lncRNAs. Biochem. Biophys. Res. Commun. 811, 153561. doi: 10.1016/j.bbrc.2026.153561 41797182

[B15] Eladl O. Yamaoki Y. Nagata T. Katahira M. (2025). In-cell NMR spectra of chemically unmodified RNA at physiological temperature with extended lifetime through RNase inhibitor cocktail in living human cells. Sci. Rep. 15, 36397. doi: 10.1038/s41598-025-20519-3 41107360 PMC12534491

[B16] Ferreira R. B. R. Antunes L. C. M. Sal-Man N. (2025). Pathogen-pathogen interactions during co-infections. ISME J. 19, wraf104. doi: 10.1093/ismejo/wraf104 40407166 PMC12145878

[B17] Gardeazabal L. Izeta A. (2024). Elastin and collagen fibres in cutaneous wound healing. Exp. Dermatol. 33, e15052. doi: 10.1111/exd.15052 38483134

[B18] Girdhar M. Sen A. Nigam A. Oswalia J. Kumar S. Gupta R. (2024). Antimicrobial peptide-based strategies to overcome antimicrobial resistance. Arch. Microbiol. 206, 411. doi: 10.1007/s00203-024-04133-x 39311963

[B19] Han S. Yang J. Qiu Y. Zhao S. Jiang Y. Han L. . (2026). lncRNA-803 suppresses apoptosis in DF-1 cells via the miR-6555-3p/MDM4/p53 axis. Genes (Basel). 17, 440. doi: 10.3390/genes17040440 42074558 PMC13116624

[B20] Han S. Zhao S. Ren H. Jiao Q. Wu X. Hao X. . (2024). Novel lncRNA 803 related to Marek's disease inhibits apoptosis of DF-1 cells. Avian Pathol. 53, 229–241. doi: 10.1080/03079457.2024.2316817 38323582

[B21] Hashem H. R. Amin B. H. Yosri M. (2023). Investigation of the potential roles of adipose stem cells and substances of natural origin in the healing process of E. coli infected wound model in rats. Tissue Cell. 85, 102214. doi: 10.1016/j.tice.2023.102214 37690258

[B22] Hassanshahi A. Moradzad M. Ghalamkari S. Fadaei M. Cowin A. J. Hassanshahi M. (2022). Macrophage-mediated inflammation in skin wound healing. Cells. 11, 2953. doi: 10.3390/cells11192953 36230913 PMC9564023

[B23] Huang J. Z. Chen M. Chen D. Gao X. C. Zhu S. Huang H. . (2017). A peptide encoded by a putative lncRNA HOXB-AS3 suppresses colon cancer growth. Mol. Cell 68, 171–184.e176. doi: 10.1016/j.molcel.2017.09.015 28985503

[B24] Hurlow J. Wolcott R. D. Bowler P. G. (2025). Clinical management of chronic wound infections: The battle against biofilm. Wound Repair Regen. 33, e13241. doi: 10.1111/wrr.13241 39600232

[B25] Ji S. An F. Zhang T. Lou M. Guo J. Liu K. . (2024). Antimicrobial peptides: An alternative to traditional antibiotics. Eur. J. Med. Chem. 265, 116072. doi: 10.1016/j.ejmech.2023.116072 38147812

[B26] Juretić D. Simunić J. (2019). Design of α-helical antimicrobial peptides with a high selectivity index. Expert Opin. Drug Discov. 14, 1053–1063. doi: 10.1080/17460441.2019.1642322 31311351

[B27] Kovaleva O. V. Rashidova M. A. Sinyov V. V. Malashenko O. S. Gratchev A. (2025). M1 macrophages - unexpected contribution to tumor progression. Front. Immunol. 16, 1638102. doi: 10.3389/fimmu.2025.1638102 40821768 PMC12350380

[B28] Li C. Wang X. Rao J. Zeng Y. Liu J. Tang F. (2024). Investigating the distribution and antibiotic resistance of bacterial pathogens in clinical specimens from a Chinese maternal and child hospital: The role of environmental factors. Infect. Drug Resist. 17, 2261–2272. doi: 10.2147/idr.S468419 38854782 PMC11162237

[B29] Li Y. Zhang J. Qi C. Mo Q. Zhong K. Liu J. . (2025). lncRNA-encoded small peptide promotes viral infection. Mol. Plant Pathol. 26, e70084. doi: 10.1111/mpp.70084 40242941 PMC12004088

[B30] Liu W. Yu M. Xie D. Wang L. Ye C. Zhu Q. . (2020). Melatonin-stimulated MSC-derived exosomes improve diabetic wound healing through regulating macrophage M1 and M2 polarization by targeting the PTEN/AKT pathway. Stem Cell Res. Ther. 11, 259. doi: 10.1186/s13287-020-01756-x 32600435 PMC7322868

[B31] Liu Y. Long S. Wang H. Wang Y. (2024). Biofilm therapy for chronic wounds. Int. Wound J. 21, e14667. doi: 10.1111/iwj.14667 38339793 PMC10858329

[B32] Louiselle A. E. Niemiec S. M. Zgheib C. Liechty K. W. (2021). Macrophage polarization and diabetic wound healing. Transl. Res. 236, 109–116. doi: 10.1016/j.trsl.2021.05.006 34089902

[B33] Luo M. Zhao F. Cheng H. Su M. Wang Y. (2024). Macrophage polarization: An important role in inflammatory diseases. Front. Immunol. 15, 1352946. doi: 10.3389/fimmu.2024.1352946 38660308 PMC11039887

[B34] Lyu Z. Y. Zhen J. H. Meng Q. Y. Zhou W. An J. Y. Dong F. (2023). Bacterial etiology and antimicrobial resistance pattern of pediatric bloodstream infections in Beijing 2015-2019. Infect. Drug Resist. 16, 6297–6308. doi: 10.2147/idr.S426000 37780532 PMC10540788

[B35] Mani R. Holmes J. Rerkasem K. Papanas N. (2023). Blood vessel density measured using dynamic optical coherence tomography is a tool for wound healers. Int. J. Low Extrem Wounds. 22, 235–240. doi: 10.1177/15347346211017334 33960852

[B36] Maron B. Rolff J. Friedman J. Hayouka Z. (2022). Antimicrobial peptide combination can hinder resistance evolution. Microbiol. Spectr. 10, e0097322. doi: 10.1128/spectrum.00973-22 35862981 PMC9430149

[B37] Mende K. Akers K. S. Tyner S. D. Bennett J. W. Simons M. P. Blyth D. M. . (2022). Multidrug-resistant and virulent organisms trauma infections: Trauma infectious disease outcomes study initiative. Mil. Med. 187, 42–51. doi: 10.1093/milmed/usab131 35512375 PMC9278338

[B38] Meng H. Su J. Shen Q. Hu W. Li P. Guo K. . (2025). A smart MMP-9-responsive hydrogel releasing M2 macrophage-derived exosomes for diabetic wound healing. Adv. Healthc Mater. 14, e2404966. doi: 10.1002/adhm.202404966 39955735

[B39] Núñez-Martínez H. N. Recillas-Targa F. (2022). Emerging functions of lncRNA loci beyond the transcript itself. Int. J. Mol. Sci. 23, 6258. doi: 10.3390/ijms23116258 35682937 PMC9181104

[B40] Rahim K. Saleha S. Zhu X. Huo L. Basit A. Franco O. L. (2017). Bacterial contribution in chronicity of wounds. Microb. Ecol. 73, 710–719. doi: 10.1007/s00248-016-0867-9 27742997

[B41] Raja S. R. Sk M. H. Wajeed S. Yangdol R. Yadav A. Jindal H. . (2025). Regulation of macrophage transcriptional dynamics during acute and chronic wound repair. Inflamm. Res. 74, 164. doi: 10.1007/s00011-025-02133-1 41236567

[B42] Ruhal R. Kataria R. (2021). Biofilm patterns in gram-positive and gram-negative bacteria. Microbiol. Res. 251, 126829. doi: 10.1016/j.micres.2021.126829 34332222

[B43] Russell S. K. Harrison J. K. Olson B. S. Lee H. J. O'Brien V. P. Xing X. . (2023). Uropathogenic Escherichia coli infection-induced epithelial trained immunity impacts urinary tract disease outcome. Nat. Microbiol. 8, 875–888. doi: 10.1038/s41564-023-01346-6 37037942 PMC10159856

[B44] Schuster R. Younesi F. Ezzo M. Hinz B. (2023). The role of myofibroblasts in physiological and pathological tissue repair. Cold Spring Harb. Perspect. Biol. 15, a041231. doi: 10.1101/cshperspect.a041231 36123034 PMC9808581

[B45] Song P. Zhao L. Zhu L. Sha G. Dong W. (2023). BsR1, a broad-spectrum antibacterial peptide with potential for plant protection. Microbiol. Spectr. 11, e0257823. doi: 10.1128/spectrum.02578-23 37948344 PMC10714738

[B46] Sorg H. Sorg C. G. G. (2023). Skin wound healing: Of players, patterns, and processes. Eur. Surg. Res. 64, 141–157. doi: 10.1159/000528271 36417847

[B47] Xiang X. Fu Y. Zhao K. Miao R. Zhang X. Ma X. . (2021). Cellular senescence in hepatocellular carcinoma induced by a long non-coding RNA-encoded peptide PINT87aa by blocking FOXM1-mediated PHB2. Theranostics. 11, 4929–4944. doi: 10.7150/thno.55672 33754036 PMC7978318

[B48] Xiao G. Li J. Sun Z. (2023). The combination of antibiotic and non-antibiotic compounds improves antibiotic efficacy against multidrug-resistant bacteria. Int. J. Mol. Sci. 24, 15493. doi: 10.3390/ijms242015493 37895172 PMC10607837

[B49] Xu W. Yao Y. Jia Y. Jiang L. Li L. Pan Y. . (2025). Antimicrobial resistance profiles of bacterial conjunctivitis isolates from a secondary hospital in Shanghai: A 5-year retrospective study, (2020-2024). Infect. Drug Resist. 18, 6779–6787. doi: 10.2147/idr.S562024 41457997 PMC12743327

[B50] Xue Y. Reddy S. K. Garza L. A. (2022). Toward understanding wound immunology for high-fidelity skin regeneration. Cold Spring Harb. Perspect. Biol. 14, a041241. doi: 10.1101/cshperspect.a041241 35667792 PMC9248820

[B51] Yao S. Hao L. Zhou R. Jin Y. Huang J. Wu C. (2022). Multispecies biofilms in fermentation: Biofilm formation, microbial interactions, and communication. Compr. Rev. Food Sci. Food Saf. 21, 3346–3375. doi: 10.1111/1541-4337.12991 35762651

[B52] Yi Q. Feng J. Lan W. Shi H. Sun W. Sun W. (2024). CircRNA and lncRNA-encoded peptide in diseases, an update review. Mol. Cancer 23, 214. doi: 10.1186/s12943-024-02131-7 39343883 PMC11441268

[B53] Yin X. Li Q. McNutt P. M. Zhang Y. (2022). Urine-derived stem cells for epithelial tissues reconstruction and wound healing. Pharmaceutics 14, 1669. doi: 10.3390/pharmaceutics14081669 36015295 PMC9415563

[B54] Younesi F. S. Miller A. E. Barker T. H. Rossi F. M. V. Hinz B. (2024). Fibroblast and myofibroblast activation in normal tissue repair and fibrosis. Nat. Rev. Mol. Cell Biol. 25, 617–638. doi: 10.1038/s41580-024-00716-0 38589640

[B55] Zhang Y. (2024). LncRNA-encoded peptides in cancer. J. Hematol. Oncol. 17, 66. doi: 10.1186/s13045-024-01591-0 39135098 PMC11320871

[B56] Zhao R. Liang H. Clarke E. Jackson C. Xue M. (2016). Inflammation in chronic wounds. Int. J. Mol. Sci. 17, 2085. doi: 10.3390/ijms17122085 27973441 PMC5187885

[B57] Zhou S. Zhang M. Xue L. Li J. Ma X. Zheng Z. . (2025). Dopamine-alginate-zinc ion dressings employing synergistic active and passive antimicrobial strategies for enhanced burn wound infection management and accelerated healing. Carbohydr. Polym. 359, 123571. doi: 10.1016/j.carbpol.2025.123571 40306778

[B58] Zhu G. Sun Z. Hui P. Chen W. Jiang X. (2021). Composite film with antibacterial gold nanoparticles and silk fibroin for treating multidrug-resistant E. coli-infected wounds. ACS Biomater. Sci. Eng. 7, 1827–1835. doi: 10.1021/acsbiomaterials.0c01271 33966376

